# Word Recall: Cognitive Performance Within Internet Surveys

**DOI:** 10.2196/mental.3969

**Published:** 2015-06-02

**Authors:** Shannon K Runge, Benjamin M Craig, Heather S Jim

**Affiliations:** ^1^ University of South Florida and Moffitt Cancer Center Tampa, FL United States; ^2^ Moffitt Cancer Center and University of South Florida Tampa, FL United States

**Keywords:** cognition, online surveys, episodic memory, Health and Retirement Study, Women’s Health Valuation Study

## Abstract

**Background:**

The use of online surveys for data collection has increased exponentially, yet it is often unclear whether interview-based cognitive assessments (such as face-to-face or telephonic word recall tasks) can be adapted for use in application-based research settings.

**Objective:**

The objective of the current study was to compare and characterize the results of online word recall tasks to those of the Health and Retirement Study (HRS) and determine the feasibility and reliability of incorporating word recall tasks into application-based cognitive assessments.

**Methods:**

The results of the online immediate and delayed word recall assessment, included within the Women’s Health and Valuation (WHV) study, were compared to the results of the immediate and delayed recall tasks of Waves 5-11 (2000-2012) of the HRS.

**Results:**

Performance on the WHV immediate and delayed tasks demonstrated strong concordance with performance on the HRS tasks (ρc=.79, 95% CI 0.67-0.91), despite significant differences between study populations (*P*<.001) and study design. Sociodemographic characteristics and self-reported memory demonstrated similar relationships with performance on both the HRS and WHV tasks.

**Conclusions:**

The key finding of this study is that the HRS word recall tasks performed similarly when used as an online cognitive assessment in the WHV. Online administration of cognitive tests, which has the potential to significantly reduce participant and administrative burden, should be considered in future research studies and health assessments.

## Introduction

The use of Internet-enabled devices, such as computers, smartphones, and tablets, to conduct cognitive research has increased dramatically over the past decade [1-3]. These devices allow researchers to use application-based cognitive assessments that have distinct advantages over more traditional assessment methods (ie, face-to-face interviews), including rapid data collection, reduced participant and administrative burden, and access to diverse or hard-to-reach populations [4,5]. When used in either a community or clinic setting, such online applications may detect cognitive and behavioral information that is missed with face-to-face assessments [6], including millisecond changes in cognitive processes [2]. Furthermore, in light of recent recommendations that cognitive screenings be included as a part of routine personalized health care [7], online cognitive assessments may play an important role in detecting subtle changes in cognitive function for both healthy and clinical populations at times when prevention and intervention strategies may have an optimal impact [8].

Application-based administration of cognitive tests has the potential to significantly advance research examining changes in cognition due to aging or illness. Repeated, short online cognitive batteries can provide a fine-grained assessment of cognitive capabilities in everyday life. For example, studies could examine situations or times of day in which cognitive lapses are most likely to occur (ie, during stress) [9,10], which can be used to devise targeted behavioral interventions to improve cognition. Similarly, more frequent cognitive assessments may help to better understand patterns of cognitive change over time in research cohorts or clinical settings.

Frequent use of cognitive assessments may be particularly important in clinical and primary care settings, where early indicators of mild cognitive impairment can be misdiagnosed as typical age-related declines in as many as 91% of cases [11]. This rate of misdiagnosis may be attributable to the frequent use of the Mini-Mental Status Examination, which lacks sensitivity to detect subclinical levels of cognitive decline compared to other assessments [12,13]. Rates of misdiagnosis are further exacerbated by individual subjective memory complaints [14]. Measures that evaluate more specific cognitive domains like episodic memory may be more specific for the detection of early changes in cognitive performance.

Episodic memory is one of the first domains in which people experience subclinical changes in cognitive performance [15,16]. Broadly described as a person’s ability to recall temporally related events or dates [17], episodic memory is particularly sensitive to the effects of aging [18-20]. This is likely a reflection of age-related neurobiological changes that occur in areas of the brain associated with episodic memory (eg, prefrontal cortex, medial temporal lobes, and hippocampus [19,21,22]), such as the decreased availability of the neurotransmitter dopamine [23], changes in functional connectivity between brain regions [24,25], and volumetric reductions of the hippocampus and prefrontal cortex [21].

Recent evidence indicates that subtle changes in episodic memory can be detected in individuals with normal or slightly impaired cognitive abilities [26]. Examining episodic memory in clinical or research settings may be particularly valuable since lower baseline scores and greater rates of changes in episodic memory are likely to precede the onset of clinical symptoms of cognitive decline [16,27], especially for individuals with a genetic risk for Alzheimer’s disease [28]. Recall tests are frequently used to estimate episodic memory as a part of larger interview-based [26,29] and online [3] neuropsychological batteries. Despite the clear advantages and potential benefits of application-based cognitive assessments, researchers often fail to demonstrate equivalence between their application-based assessment and its interview-based counterpart [3]. Ideally, equivalence between assessments (ie, construct validity) would be evaluated using a gold standard measure [30]. In the absence of such a standard, it is preferable to use an internally consistent and valid measure that has demonstrated response stability across samples [31,32].

In response to this gap, the current study opted to replicate the episodic memory tasks (immediate and delayed recall) of the Health and Retirement Study (HRS) in an online survey. These tasks were selected for a number of reasons. First, performance on the cognitive measures of the HRS has shown to be stable from wave to wave, after controlling for cohort effects and test-retest bias [33]. Second, none of these measures has been adapted for use in application-based assessments and tested for equivalence. Third, the format and presentation of the episodic tasks of the HRS were most easily replicated in an online format and would not require the use of complex computer technology that may be difficult or unavailable for older populations (eg, microphones). Finally, due to the authors’ interest in age, this study was further motivated by evidence that episodic memory is more susceptible to increasing age compared to semantic memory (ie, abilities related to vocabulary and general knowledge [34]), which has been shown to remain stable well into later decades of life [18,20]. Given the age range of the online sample in the current study (40-69 years), as well as the previous methodological considerations, the replication of the episodic memory tasks was prioritized higher than the other HRS measures.

This study examines the performance of an online word recall task that was originally developed as part of the HRS for cognitively healthy adults. Specifically, the results of an online immediate and delayed word recall task in a nationally representative sample of women aged 40 to 69 years were compared to the results of female respondents from waves 5-11 (2000-2012) of the HRS. Using these primary and secondary data, two questions were examined: (1) Do the online word recall tasks demonstrate sufficient equivalence to the HRS word recall tasks? (2) Does word recall performance vary as a function of respondent characteristics and task modality? Ultimately, the results of this study will aid in the evaluation of the potential of cognitive assessments in online surveys and health assessments.

## Methods

### Study Samples

#### The Health and Retirement Study

Since its launch in 1992, the goal of the Health and Retirement Study (HRS) has been to provide a detailed, national representation of US adults aged 50 years and older. Jointly managed through the National Institute on Aging (U01 AG009740), the Institute for Social Research, and the University of Michigan (IRB Protocols HUM00056464, HUM00061128, HUM00002562, HUM00079949, HUM00080925, and HUM00074501), the HRS is widely cited as an excellent source of data for use in examining cognitive trends and abilities of the aging US population [35]. Data is collected via telephone and face-to-face interviews in 2-year cycles, with new cohorts added every 6 years. The HRS uses a dual modality approach, where initial interviews are conducted face-to-face and the majority of successive interviews are conducted over the telephone (unless participants are older than 80 years of age). Hispanic and black adults are oversampled. Spouses of HRS participants are also included, regardless of age.

#### The Women’s Health Valuation Study

Conducted at Moffitt Cancer Center in Tampa, Florida, the Women’s Health Valuation (WHV) study is an Internet-based health valuation study that included health measures and a discrete choice experiment (DCE) where respondents reported their preferences between possible health outcomes. The approach and methods, including its sampling design and survey instrument, were adapted from the PROMIS-29 valuation study (1R01CA160104) [36] and approved by the University of South Florida Institutional Review Board (USF IRB Protocol 8236).

The WHV online survey instrument had four components: screener, health, DCE, and follow-up. Each component had a series of questions distributed across a continuous series of pages, and responses were recorded by clicking or typing answers and then hitting the *Next* button. Each page included a *Back* button so the respondent could return to previous pages and change previous answers; however, to discourage participants from returning to previous pages of the survey, the *Back* button was disabled. To exit the survey, respondents could close their browser at any time. If the browser was closed prior to completing the survey, the data were not recorded. Responses to all questions were mandatory in order to proceed to the next page.

Participants were recruited from a pre-existing national panel of US adults. To promote concordance with the 2010 US Census, participants were sampled according to 6 demographic quotas: age in years (40-54 and 55-69) and race/ethnicity (Hispanic; black, non-Hispanic; white; and other, non-Hispanic). Further details about the methods of this study are available online [37]. Overall, 4474 women completed the survey between April 3, 2013 and April 21, 2013.

### Cognitive Measures of the Health and Retirement Study

#### Episodic Memory

The cognitive battery of the HRS has been evaluated for internal consistency and validity [38]. Latent factor path modeling has identified three cognitive domains: episodic memory (immediate and delayed recall), mental status (serial 7s, backward counting from 20, naming), and vocabulary (ie, semantic memory) [35]. Measures of episodic memory include an immediate and delayed recall task. Mental status is measured by a serial 7s subtraction test, counting backwards from 20, and naming (the last name of the current president and vice president; two objects [scissors and cactus] based on a brief verbal description; and the current month, day, year, and day of week). Semantic memory is assessed using a baseline measure of vocabulary (5 words) [39].

As a measure of episodic memory, the immediately and delayed recall tasks are drawn from four categorized lists of 10 English nouns that did not overlap in content. Respondents are randomly assigned to one of the four lists at the initial interview. Longitudinally, each respondent is randomly assigned to receive an alternative word list, such that each respondent is assigned to a different set of words for the three successive waves of data collection. With this counterbalanced approach, each respondent was assigned to each word list only once over 4 waves of data collection, and approximately 8 years will pass before a respondent is reassigned to the same set of words as their initial interview.

During the immediate recall task, an interviewer reads a list of 10 words at a rate of approximately 2 seconds per word to each respondent, who verbally recalled as many words as possible. Approximately 5 minutes after the immediate word recall test, during which respondents answered questions about their emotional state and completed two mental status tasks (eg, counting backwards, serial 7s), respondents were asked to recall the words from the immediate recall task. For each task, the number of correctly recalled words is scored, with higher scores indicating better performance.

#### Self-Reported Memory

In addition to episodic memory, HRS respondents are also asked to self-report their memory at the present time (excellent, very good, good, fair, or poor) and compare their current memory to their memory 2 years ago (better, same, or worse).

For the purpose of comparison, this study examines all word recall responses from waves 5-11 (2000-2012) of the HRS. Since the WHV was restricted to female respondents, we excluded male respondents from the HRS to decrease the risk of gender bias. Participants of the HRS who reported using a proxy respondent; refused to respond to word recall tasks; or had missing data on demographic, memory, or word recall variables (less than 2.0% of the sample) were also excluded. Aside from these exclusion criteria, 12,545 women completed between 1 and 7 word recall tasks with a median (interquartile range) of 3 tasks (2-5 tasks). These tasks were restructured to represent a cross-sectional dataset with a total of 43,417 word recall tasks.

### Cognitive Measures of the Women’s Health Valuation Study

#### Episodic Memory

The episodic memory of the WHV replicated the word recall task conducted as part of the HRS. All respondents were asked to recall 10 English nouns immediately after they were presented on-screen (immediate recall) and after a delay (delayed recall). Each respondent received one of four randomly assigned sets of words, which were taken verbatim from the HRS and presented in the same order. Prior to the immediate recall task, respondents were presented with a screen that informed them that they would be shown a set of 10 words and would be asked to recall as many words as they could. These instructions were largely based on those given to HRS respondents but modified for online presentation. Words appeared on the computer screen one at a time for approximately 3 seconds. Respondents were asked to recall the words directly after the presentation of all 10 words (immediate recall) and then approximately 20 minutes later at the end of the DCE component (delayed recall). For each recall, respondents typed as many words as they could remember, in any order, in empty text boxes within the survey. As with the HRS, the primary measure of episodic memory was the sum of correctly recalled words for each task, regardless of order.

#### Self-Reported Memory

The self-reported memory questions of the WHV were replicated from the self-reported memory questions of the HRS. As part of the health component, the self-reported memory questions asked participants to rate their memory at the present time (excellent, very good, good, fair, or poor) and compare their current memory to their memory 2 years ago (better, same, or worse).

Compared to the word recall task in the HRS, the online task in WHV differed in the several ways. The word lists were displayed visually on a computer device/browser as opposed to being spoken by an interviewer (basic literacy skills were required, with less reliance on verbal communication), respondents recalled words by typing them versus speaking them (basic typing skills were required, with less reliance on verbal communication), and the words can sound the same with different spelling (eg, see vs sea and rock vs roc), which may make the WHV task more specific. In addition, the delay between the immediate and delayed recalls task was shorter (5 minutes vs 20 minutes) and the WHV version was purely cross-sectional, whereas HRS respondents may have completed the tasks up to seven times. Nevertheless, the study took all available steps possible to replicate the original HRS tasks.

### Statistical Analyses

Demographic and descriptive statistics (Table 1) obtained on both groups were analyzed using independent sample *t* tests, Pearson chi-square, and one-way analyses of variance, where appropriate. In order to estimate the precision and accuracy of the two word recall tasks, Lin’s concordance correlation coefficient (*ρ*_*c*_) [30] was used to collectively compare the average frequency with which the WHV and HRS participants recalled each word. Unlike Pearson’s correlation coefficient, which estimates only the linear covariation between variables, Lin’s concordance quantifies the degree of agreement between two measures of the same variable by providing a measure of covariation and correspondence [30]. Finally, multivariate linear regression models adjusted for cluster errors (ie, multiple tasks per respondent) were used to estimate the associations between characteristics of each study sample and number of correctly recalled words for the immediate and delayed recall tasks. All analyses were conducted using Stata 13 software (StataCorp).

## Results

### Overview

The WHV online survey had 4474 respondents, each of whom completed 1 word recall task. The HRS survey had 12,545 respondents who completed between 1 and 7 recall tasks. As shown in Table 1, WHV respondents differed significantly from HRS respondents along each characteristic. Overall, WHV respondents were more likely to be white or Hispanic, younger, and better educated and report excellent or very good memory compared to HRS respondents, possibly due to sampling from an online panel.

**Figure 1:**
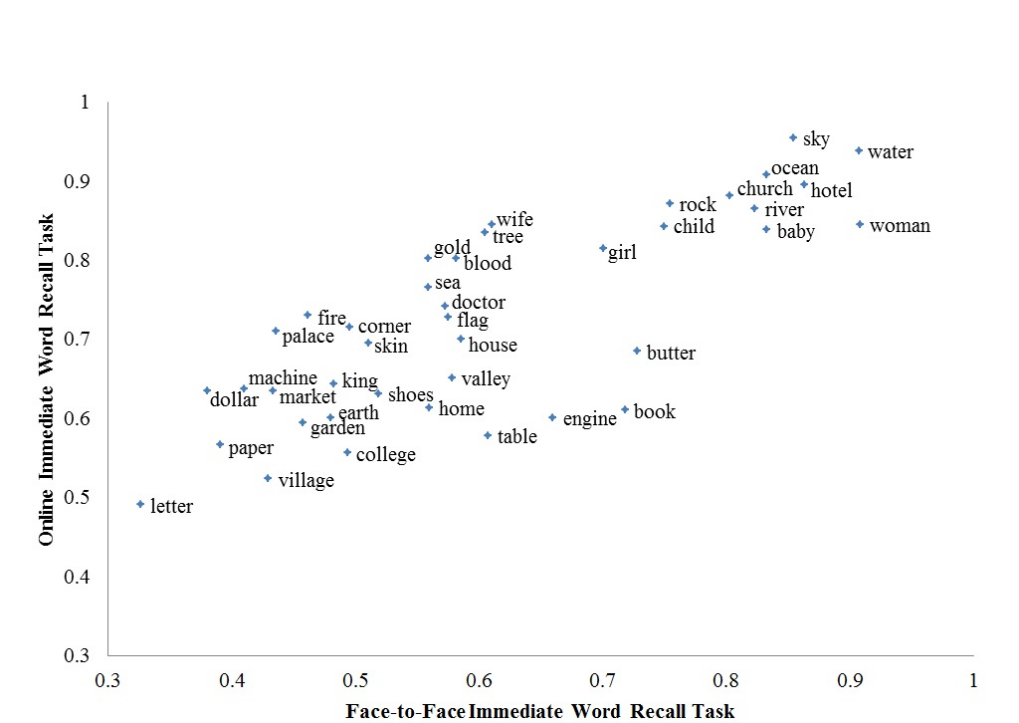
Likelihood of immediate recall by word.

Figure 1 is a scatterplot of the likelihood of immediate recall for each word by modality, which ranges from 0.49 to 0.85 for WHV respondents and 0.33 to 0.91 for HRS respondents. Out of the 40 words, 35 words had greater recall for the WHV versus HRS task with a mean difference of 11.82% (95% CI −0.31 to 0.08). At first glance, Lin’s concordance correlation coefficient (*ρ*_*c*_=.57, 95% CI 0.42-0.722) indicated mild correspondence. Once the likelihoods were normalized (ie, subtracting the sample mean and dividing by the standard deviation), Lin’s concordance correlation coefficient increased to .789 (95% CI 0.67-0.91), indicating strong correspondence. Similarly, the delayed recall task showed Lin’s concordance correlation coefficient with and without normalization that suggested strong concordance (*ρ*_*c*_=.82, 95% CI 0.72-0.91 and *ρ*_*c*_=.86, 95% CI 0.76-0.94, respectively; not shown).

For the immediate and delayed recall tasks, this study assessed differences in association between the number of correctly recalled words by study sample and word list assignment (Table 2), as well as sociodemographic differences between samples (Table 3). Results from the regression analyses were interpreted using a base scenario that represents the median sociodemographic characteristics of the sample (ie, the average number of words that are correctly recalled by a white female aged 50-54 years who is married, has a high school diploma, and self-reports her current memory as good). For immediate or delayed recall, WHV respondents recalled significantly more words than HRS respondents, except for List 3 in delayed recall. For both WHV and HRS respondents, the number of correctly recalled words varied significantly depending on which list was assigned; however, these differences were small (<0.28 words).

**Table 1:** Respondent characteristics by modality.

		WHV	HRS	*P* value
Number of respondents		4474	12,545	
Number of tasks per respondent, median (IQR)		1	3 (2-5)	
Total number of tasks		4474	43,417	
**Age in years, median (IQR)**		53 (48-61)	60 (55-64)	<.001
	40-44, n (%)	629 (14.17)	735 (1.68)	
	45-49, n (%)	754 (16.98)	2158 (4.94)	
	50-54, n (%)	1051 (23.67)	7701 (17.63)	
	55-59, n (%)	661 (14.89)	10, 951 (25.07)	
	60-64, n (%)	641 (14.44)	11,260 (25.78)	
	65-69, n (%)	704 (15.86)	10,868 (24.88)	
**Race**				<.001
	White, n (%)	3556 (80.09)	33,992 (75.14)	
	Black, n (%)	632 (14.23)	8832 (19.52)	
	Other, n (%)	252 (5.68)	2412 (5.33)	
**Hispanic ethnicity**				<.001
	No, n (%)	3743 (84.30)	39,604 (87.27)	
	Yes, n (%)	697 (15.70)	5774 (12.72)	
**Educational attainment**				<.001
	No degree, n (%)	168 (3.78%)	8334 (18.40)	
	High school diploma/GED, n (%)	1955 (44.03)	24,941 (55.05)	
	Associates degree/some college, n (%)	1257 (28.31)	2747 (6.06)	
	Bachelor's degree, n (%)	669 (15.07)	5664 (12.50)	
	Master's degree, n (%)	320 (7.21)	3198 (7.06)	
	Law/MD/PhD, n (%)	71 (1.60)	419 (0.92)	
**Marital status**				<.001
	Married, n (%)	2338 (53.78)	28,333 (62.35)	
	Partnered, n (%)	231 (5.20)	2069 (4.55)	
	Separated/divorced, n (%)	1048 (23.60)	8654 (19.04)	
	Widowed, n (%)	262 (5.90)	4922 (10.83)	
	Never married, n (%)	511 (11.51)	1467 (3.23)	
**Self-reported current memory**				<.001
	Excellent, n (%)	468 (10.54)	2518 (5.77)	
	Very good, n (%)	1743 (39.04)	11,353 (26.00)	
	Good, n (%)	1704 (38.38)	18,991 (43.48)	
	Fair, n (%)	486 (10.93)	9137 (20.92)	
	Poor, n (%)	48 (1.08)	1654 (3.79)	

**Table 2:** Average number of correctly recalled words by list and modality.

	Immediate recall			Delayed recall		
	WHV	HRS	*P* value^a^	WHV	HRS	*P* value^a^
Overall	7.24	6.06	1	5.41	5.21	<.001
List 1^a^	7.40	6.14	.<001	5.57	5.24	<.001
List 2^a^	7.16	5.93	.<001	5.29	5.08	.013
List 3^a^	7.12	6.12	<.002	5.27	5.28	.922
List 4^a^	7.30	6.07	<.001	5.50	5.23	.002

^a^Significant differences were detected between lists for immediate (*P*_*WHV*_ < .001 and *P*_*HRS*_ < .001) and delayed (*P*_*WHV*_ =.002 and *P*_*HRS*_ < .001) word recall tasks.

**Table 3:** Associated respondent characteristics and number of correctly recalled words by survey modality: WHV versus HRS.

		Immediate recall			Delayed recall		
		WHV	HRS	*P* value^a^	WHV	HRS	*P* value^a^
Constant^b^		7.19^d^	6.34^d^	<.001	5.62^d^	5.48^d^	<.001
**Age in years**							
	40-44	−.04	.25^d^	.01	−.13	.31^d^	<.001
	45-49	−.01	.01	.84	−.05	.01	.65
	50-54	—	—	—	—	—	—
	55-59	.14	−.01	.10	−.04	.04	.57
	60-64	.01	−.07^d^	.48	−.10	.02	.39
	65-69	.02	−.24^d^	<.001	.15	−.16^d^	.03
**Race**							
	White	—	—	—	—	—	—
	Black	−.55^d^	−.50^d^	.57	−.71^d^	−.74^d^	.86
	Other	−.16	−.34^d^	.26	−.32	−.41^d^	.70
**Hispanic ethnicity**							
	No	—	—	—	—	—	—
	Yes	−.19^c^	−.36^d^	.08	−.10	−.35^d^	.09
**Educational attainment**							
	No degree	−.36^c^	−.61^d^	.14	−.25	−.64^d^	.07
	High school diploma/GED	—	—	—	—	—	—
	Associate’s degree/ some college	.12	.19^d^	.35	−.13	.18^d^	<.001
	Bachelor's degree	.35^d^	.47^d^	.19	.26^c^	.50^d^	.07
	Master's degree	.60^d^	.61^d^	.92	.40^c^	.68^d^	.12
	Law/MD/PhD	.67^d^	1.01^d^	.13	.52	1.09^d^	.16
**Marital**							
	Married	—	—	—	—	—	—
	Partnered	−.14	−.10^d^	.79	.17	−.11^c^	.17
	Separated/divorced	−.02	−.07^d^	.45	−.17	−.10^d^	.52
	Widowed	.04	−.04	.48	.05	−.03	.67
	Never married	.05	−.17^d^	.04	−.05	−.14^c^	.56
**Self-reported current memory**							
	Excellent	.18	.13^d^	.64	.12	.12^d^	.99
	Very good	.22^d^	.21^d^	.97	.07	.24^d^	.10
	Good	—	—	—	—	—	—
	Fair	−.52^d^	−.31^d^	.03	−.60^d^	−.35^d^	<.001
	Poor	−1.53^d^	−.74^d^	.01	−1.93^d^	−.85^d^	<.001

^a^Represents *P* value for H^0^: No difference between online and face-to face.

^b^Base scenario represents the average number of words that are correctly recalled by a white female, aged 50-54 years, who is married, has a high school diploma, and self-reports her current memory as good.

^c^ *P* value <.05.

^d^ *P* value <.01.

### Immediate Word Recall

Immediate word recall was significantly associated with respondent characteristics in WHV and HRS tasks, and there were significant modality differences between the online and HRS studies. Overall, WHV respondents immediately recalled about one more word (0.85) than HRS respondents did, after adjusting for respondent characteristics. In terms of demographics, age was significantly associated with immediate recall for the HRS task but not the WHV task. Specifically, younger respondents recalled more words than older respondents in the HRS tasks but not in the WHV tasks. Non-white and/or Hispanic respondents were significantly associated with reduced immediate recall for either modality; however, their associations were not significantly different by modality.

Levels of educational attainment were significantly associated with immediate recall for both the HRS and WHV tasks. Detrimental effects were seen for the lowest education level; respondents with less than a high school diploma recalled fewer words. The benefits of obtaining education beyond high school were incrementally significant, with the exception of WHV respondents who earned an associate’s degree. Marital status was significantly associated with immediate recall in the HRS tasks but not the WHV tasks. Specifically, respondents who reported being partnered, separated, divorced, or never married recalled fewer words than their married counterparts. However, the only associations that differed significantly between modalities were those for individuals who were never married.

Self-reported current memory was significantly associated with immediate word recall in both modalities. As expected, those who reported their memory as excellent or very good were more likely to recall more words than those with a fair or poor memory. However, it is unclear whether those who reported excellent memory had better recall than those who reported very good memory. The association between a poor memory and immediate word recall was statistically significant with a noteworthy effect (1.53 words less than good memory). The association with fair or poor was greater for the WHV task than the HRS task, possibly because of interviewer biases (eg, slowing the task for persons who reported poor memory).

### Delayed Word Recall

As with immediate word recall, the associations between respondent characteristics and delayed word recall were significant, and their associations differed by modality. Adjusting for respondent characteristics, WHV respondents recalled approximately 0.14 more words after a delay than HRS respondents. Like the immediate recall results, the association between age and delayed recall was significant for the HRS task but not the WHV task. For both modalities, respondents who were Non-white and/or Hispanic performed significantly worse on the delayed recall tasks, but the associations did not differ significantly.

Levels of educational attainment were significantly associated for both modalities and differed slightly from what was seen for the immediate recall task. Significant detrimental effects were no longer seen for WHV respondents with less than a high school diploma but persisted for HRS respondents. Higher levels of education beyond an associate’s degree remained significantly associated with greater delayed recall, with the exception of WHV respondents who earned an associate’s or advanced degree. The association between advanced education levels and recall was very strong for HRS respondents, who recalled approximately 0.50 more words compared to similarly educated WHV respondents. Marital status was significantly associated with delayed recall for the HRS modality but not the online modality. HRS respondents who reported being partnered, separated or divorced, or never married recalled significantly fewer words compared to married respondents. The associations between modalities were not significantly different.

Self-reported current memory was significantly associated with delayed word recall in both modalities. Similar to the immediate recall task, respondents who reported their memory as excellent or very good were more likely to recall more words than those with a fair or poor memory. The association between poor memory and delayed recall intensified for WHV respondents, who recalled nearly 2 words less compared to the base scenario and more than 1 word less compared to HRS respondents with a similar memory rating.

In order to explore the possibility that word recall scores for WHV respondents were influenced by literacy level and typing skills (ie, misspelled words would not be counted as correct), the previous analyses were rerun after correcting words that were misspelled by one letter. This arbitrary adjustment was based on the number of WHV responses that appeared to be related to misspellings (eg, doller for dollar) or mistyping (eg, ovean for ocean), and is akin to the best-judgment practice granted to HRS interviewers when determining whether a HRS response should be counted as correct (eg, woman for women or shoe for shoes). When the analyses were rerun using the spell-corrected word counts, no significant differences were seen for any of the results. Therefore, the results reported here were conducted using the uncorrected word recall responses for WHV respondents.

## Discussion

### Principal Findings

This study compared and characterized the results of the WHV word recall task to those of a gold standard HRS word recall task in order to determine reliability for future surveys. The results of this study provide support for the inclusion of online cognitive assessments in health surveys. This is the first study attempting to replicate the HRS word recall tasks in an application-based assessment. The results indicate that the immediate and delayed word recall tasks were equivalent to the HRS tasks, as evidenced by high levels of concordance (precision) and association with self-reported memory (convergent validity). Even after controlling for age, education, and self-reported memory, WHV respondents recalled nearly one more word than HRS respondents for the immediate recall tasks. This difference decreased but remained significant for the delayed recall and may be attributed to study design differences or other unobservable sample selection biases. In summary, both HRS and WHV tasks appear to perform well despite key differences between the studies.

While our normalized results demonstrated a high level of concordance between the WHV and HRS tasks and thus support the primary goal of this study, we did note significant differences between samples that may be related to a number of potential confounders, such as differences in study design. For example, the HRS recall lists were presented verbally, whereas the words of the WHV lists were presented visually. Upon initial review, one may think that differences in how the brain processes auditory versus visual information may contribute to modality differences. However, research has shown that auditory and visual recall tasks activate overlapping regions of the brain, and while the left hemisphere of the brain is activated slightly more during visual tasks, there is no evidence that recall performance is impacted by modality [40].

An additional difference in study design is the length of time and type of activities that were completed by respondents between the immediate and delayed recall tasks. HRS respondents answered questions regarding their emotional state over the past week (eg, levels of motivation, happiness, and loneliness) and completed two mental math tasks (ie, counting backwards and subtracting 7s) for 5 minutes. WHV respondents completed a series of DCE tasks during the 20-minute delay, which may arguably require greater levels of cognitive engagement. These dissimilarities in the amount of delay and the complexity of the tasks completed during the delay may have contributed to the observed modality differences. The regression analysis may control for some of the sample selection issues, but panel and delay attributes may also explain differences by modality.

In addition to modality differences, there is a potential concern for practice effects to bias the results of repeated word recall tasks, particularly since such effects mask true declines in cognitive performance [41]. Practice effects have been associated with the cognitive data of the HRS [33,35]; however, the interpretation of these results is muddied by the complex methodology of the earliest waves of data collection. For example, Rodgers et al examined practice effects in the word recall tasks of the 1993 and 1995 waves of the Asset and Health Dynamics Among the Oldest Old Study (AHEAD) to word recall performance of the 1998 and 2000 waves of the HRS (the AHEAD and HRS were merged in 1998 due to methodological and content similarities) [33]. Although significant practice effects were identified from wave 1 (1993) to wave 2 (1995) and from wave 2 to wave 3 (1998), none were identified from wave 3 to wave 4 (2000) [33]. The authors note these results are difficult to interpret given the considerable methodological changes that were made from wave to wave, most notable of which is the implementation of the counterbalanced word recall list assignment in wave 2 of AHEAD (1995). Additionally, there is the possibility that the original word list used in 1993 was simply more difficult compared to word lists used in subsequent waves [33].

In a more recent analysis, McArdle et al found evidence of practice effects in cognitive data from earlier waves of the HRS (1992-2004) [35]; however, this result may also be affected by substantive changes in study design. Specifically, the word recall tests of 1992 and 1994 included only one word list with 20 nouns; the counterbalanced approach of randomly assigned four lists of 10 words was first implemented with the HRS in 1996. As with the results of the previous study, the presence of practice effects could be attributed to respondents receiving the same list of words in 1992 and 1994. Additionally, greater levels of recall in subsequent waves could be attributed to the fact that respondents may find it easier to recall 10 words as opposed to 20.

These methodological changes clearly restrict the interpretability of potential practice effects noted within the HRS. The results of the current study are less subjective to such biases since the analyses are restricted to the 2000-2012 waves of the HRS (ie, the counterbalanced assignment of word recall lists is uniform across waves). Despite this counterbalanced approach, it is not possible to completely rule out the potential influence of practice effects. Future studies should attempt to measure the presence and impact of practice effects in the HRS using only the waves with identical methodological approaches.

We also found several interesting associations between episodic memory performance and sociodemographic characteristics. The effect of marital status on word recall was significant only for HRS respondents; individuals who were partnered, separated or divorced, or never married performed worse compared to those who were married. The presence of significant results in the HRS sample but not the WHV sample may be related to the fact that married/partnered HRS respondents are often interviewed one after the other. Previous research has indicated that spouses who are interviewed second may be at a disadvantage in free recall tasks [42], possibly due to the fact the first interviewed spouse may be healthier. Another possible explanation of these results is that those who are partnered have been shown to perform better on episodic memory tasks in general compared to non-partnered individuals [35].

Education was another sociodemographic characteristic that was significantly associated with word recall performance, with higher levels of education significantly predicting higher episodic memory performance. Higher levels of education are thought to influence cognitive function by increasing individual levels of brain and cognitive reserve [43]. Brain reserve refers to the inherent efficiency and capability of the brain to support and execute cognitive functions [43]. Conversely, cognitive reserve represents the brain’s ability to maintain this efficiency despite the accumulation of structural and neural damage that occurs as a result of natural aging, disease, or injury [43]. Increased levels of cognitive reserve may be particularly beneficial during later stages of life [44-46]. Previous researchers have argued against controlling for the impact of education, stating that growing levels of education represent cohort trends that contribute to overall increases in cognitive performance [33]. However, it is possible that other factors associated with higher education (eg, increased socioeconomic status, better nutrition, greater availability of resources) may have attributed to this positive relationship.

While several computer-based cognitive batteries have been developed [47,48] to date, these have lacked correspondence to HRS tasks used in large cohort studies. The goal of the current study was to develop an application-based cognitive measure for episodic memory that could be easily used in future research studies and health assessments. The potential benefits of such online tasks can be inferred from evidence showing that including short cognitive tests as a part of a routine evaluation in the clinical or community setting aids in the early detection of cognitive decline. Individuals who self-report problems with memory may be more aware of adverse changes in cognitive performance [49]. Additionally, older adults who report problems with memory but perform normally have been shown to have structural brain changes similar to those seen in mild cognitive impairment [50].

### Future Research

Future research should assess additional cognitive tasks included in the HRS. This type of research might expand the results of the current study to investigate the effects of setting (eg, waiting room, hospital room, home use of online tasks) or to support the use of routine online cognitive assessments to track cognitive change in healthy older adults or clinical populations. Furthermore, clear standards for measurement using online tasks similar to the electronic patient-reported outcome literature should be created [51]. Development of such standards is likely complicated by the fact that device and software technology continues to evolve and age-related rates of cognitive change vary across a range of domains and birth cohorts with varying computer aptitudes [52,53].

### Limitations

A key limitation of the study is the use of an existing panel in the community setting. While some may argue that sampling bias is introduced by using research panels who demonstrate high levels of technological capabilities (ie, use of computers, smartphones, tablets), it has also been noted that such panels allow researchers to collect large amounts of data from diverse populations [2]. A further limitation is the lack of access to medical records that verify quality of self-reported health. Older individuals tend to rate their health more highly than younger individuals despite increases in chronic medical problems [54-56], and this overestimation of health may inadvertently bias results. The biases associated with self-reported health and behavior measures are well documented; however, expanding the current research into clinical settings would alleviate this issue. Also, the community setting adds a lack of environmental control (eg, interruptions) that may increase variability. A future project may compare interview-based and application-based tasks in a clinical population (eg, Alzheimer patients) during set times. Additionally, the current study focuses on episodic memory; in order to obtain a more robust estimation of cognitive abilities, future efforts should identify the correspondence between interview-based and online versions of other cognitive assessments of such as measures of semantic memory and vocabulary.

Inability to monitor respondent behavior is a limitation of online and telephone surveys [1]. For example, respondents of online or telephone word recall tasks could have written down the words on paper as they were presented. Examination of eye-tracking or client-side paradata [57] (ie, information about respondent behavior recorded by respondents’ computers, such as the number of times and locations of mouse clicks) has the potential to be extremely valuable in the analysis of online survey data. Nevertheless, further technological advancements are needed before such evidence can be incorporated into cognitive measures.

In summary, this study found a high level of convergent validity between the WHV and HRS word recall tasks, after controlling for age, education, and self-reported memory. Use of application-based cognitive assessments should continue to expand in community research and clinical settings, but greater efforts need to be made in regards to validating such online measures. Additionally, researchers should be wary of a number of potential biases, including modality differences, retest effects, and gender differences in cognitive performance.

## Abbreviations

DCE: discrete choice experiment
HRS: Health and Retirement Study
WHV: Women’s Health Valuation Study

## References

[1] Lee H, Baniqued PL, Cosman J, Mullen S, McAuley E, Severson J, Kramer AF (2012). Examining cognitive function across the lifespan using a mobile application. Comput Hum Behav.

[2] Dufau S, Duñabeitia JA, Moret-Tatay C, McGonigal A, Peeters D, Alario FX, Balota DA, Brysbaert M, Carreiras M, Ferrand L, Ktori M, Perea M, Rastle K, Sasburg O, Yap MJ, Ziegler JC, Grainger J (2011). Smart phone, smart science: how the use of smartphones can revolutionize research in cognitive science. PLoS One.

[3] Wild K, Howieson D, Webbe F, Seelye A, Kaye J (2008). Status of computerized cognitive testing in aging: a systematic review. Alzheimers Dement.

[4] Bohannon J (2011). Human subject research: social science for pennies. Science.

[5] Schonlau M, van Soest A, Kapteyn A, Couper M (2009). Selection bias in web surveys and the use of propensity scores. Sociol Methods Res.

[6] Parsey CM, Schmitter-Edgecombe M (2013). Applications of technology in neuropsychological assessment. Clin Neuropsychol.

[7] Borson S, Frank L, Bayley PJ, Boustani M, Dean M, Lin PJ, McCarten JR, Morris JC, Salmon DP, Schmitt FA, Stefanacci RG, Mendiondo MS, Peschin S, Hall EJ, Fillit H, Ashford JW (2013). Improving dementia care: the role of screening and detection of cognitive impairment. Alzheimers Dement.

[8] Grodstein F (2012). How early can cognitive decline be detected?. Brit Med J.

[9] Sliwinski MJ, Smyth JM, Hofer SM, Stawski RS (2006). Intraindividual coupling of daily stress and cognition. Psychol Aging.

[10] Stawski RS, Mogle J, Sliwinski MJ (2011). Intraindividual coupling of daily stressors and cognitive interference in old age. J Gerontol B Psychol Sci Soc Sci.

[11] Boustani M, Callahan CM, Unverzagt FW, Austrom MG, Perkins AJ, Fultz BA, Hui SL, Hendrie HC (2005). Implementing a screening and diagnosis program for dementia in primary care. J Gen Intern Med.

[12] Alagiakrishnan K, Zhao N, Mereu L, Senior P, Senthilselvan A (2013). Montreal Cognitive Assessment is superior to Standardized Mini-Mental Status Exam in detecting mild cognitive impairment in the middle-aged and elderly patients with type 2 diabetes mellitus. Biomed Res Int.

[13] Velayudhan L, Ryu SH, Raczek M, Philpot M, Lindesay J, Critchfield M, Livingston G (2014). Review of brief cognitive tests for patients with suspected dementia. Int Psychogeriatr.

[14] Edmonds EC, Delano-Wood L, Galasko DR, Salmon DP, Bondi MW (2014). Subjective cognitive complaints contribute to misdiagnosis of mild cognitive impairment. J Int Neuropsychol Soc.

[15] Albert M, Blacker D, Moss MB, Tanzi R, McArdle JJ (2007). Longitudinal change in cognitive performance among individuals with mild cognitive impairment. Neuropsychology.

[16] Mickes L, Wixted JT, Fennema-Notestine C, Galasko D, Bondi MW, Thal LJ, Salmon DP (2007). Progressive impairment on neuropsychological tasks in a longitudinal study of preclinical Alzheimer's disease. Neuropsychology.

[17] Tulving E, Tulving E, Donaldson E (1972). Episodic and semantic memory. Organization of Memory.

[18] Small BJ, Dixon RA, McArdle JJ, Grimm KJ (2012). Do changes in lifestyle engagement moderate cognitive decline in normal aging? Evidence from the Victoria Longitudinal Study. Neuropsychology.

[19] Tulving E (2002). Episodic memory: from mind to brain. Annu Rev Psychol.

[20] Salmon DP, Ferris SH, Thomas RG, Sano M, Cummings JL, Sperling RA, Petersen RC, Aisen PS (2013). Age and apolipoprotein E genotype influence rate of cognitive decline in nondemented elderly. Neuropsychology.

[21] Head D, Rodrigue KM, Kennedy KM, Raz N (2008). Neuroanatomical and cognitive mediators of age-related differences in episodic memory. Neuropsychology.

[22] Mayes AR, Roberts N (2001). Theories of episodic memory. Philos Trans R Soc Lond B Biol Sci.

[23] Li SC, Rieckmann A (2014). Neuromodulation and aging: implications of aging neuronal gain control on cognition. Curr Opin Neurobiol.

[24] Ford JH, Kensinger EA (2014). The relation between structural and functional connectivity depends on age and on task goals. Front Hum Neurosci.

[25] Addis DR, Leclerc CM, Muscatell KA, Kensinger EA (2010). There are age-related changes in neural connectivity during the encoding of positive, but not negative, information. Cortex.

[26] Dixon RA, de Frias CM (2014). Cognitively elite, cognitively normal, and cognitively impaired aging: neurocognitive status and stability moderate memory performance. J Clin Exp Neuropsychol.

[27] Albert M, Soldan A, Gottesman R, McKhann G, Sacktor N, Farrington L, Grega M, Turner R, Lu Y, Li S, Wang MC, Selnes O (2014). Cognitive changes preceding clinical symptom onset of mild cognitive impairment and relationship to ApoE genotype. Curr Alzheimer Res.

[28] Caselli RJ, Dueck AC, Osborne D, Sabbagh MN, Connor DJ, Ahern GL, Baxter LC, Rapcsak SZ, Shi J, Woodruff BK, Locke DEC, Snyder CH, Alexander GE, Rademakers R, Reiman EM (2009). Longitudinal modeling of age-related memory decline and the APOE epsilon 4 effect. N Engl J Med.

[29] Klekociuk SZ, Summers JJ, Vickers JC, Summers MJ (2014). Reducing false positive diagnoses in mild cognitive impairment: the importance of comprehensive neuropsychological assessment. Eur J Neurol.

[30] Lin LI (1989). A concordance correlation coefficient to evaluate reproducibility. Biometrics.

[31] Kelly PA, O'Malley KJ, Kallen MA, Ford ME (2005). Integrating validity theory with use of measurement instruments in clinical settings. Health Serv Res.

[32] Sechrest L (2005). Validity of measures is no simple matter. Health Serv Res.

[33] Rodgers WL, Ofstedal MB, Herzog AR (2003). Trends in scores on tests of cognitive ability in the elderly U.S. population, 1993-2000. J Gerontol B Psychol Sci Soc Sci.

[34] Greenberg DL, Verfaellie M (2010). Interdependence of episodic and semantic memory: evidence from neuropsychology. J Int Neuropsychol Soc.

[35] McArdle JJ, Fisher GG, Kadlec KM (2007). Latent variable analyses of age trends of cognition in the Health and Retirement Study, 1992-2004. Psychol Aging.

[36] Craig B, Schell M, Brown P, Reeve B, Cella D, Hays R, Lipscomb J, Pickard A, Revicki D (2011). HRQoL Values for Cancer Survivors: Enhancing PROMIS Measures for CER.

[37] Craig B, Owens M (2013). Methods Report of the Women's Health Valuation Study.

[38] Ofstedal MB, Fisher GG, Herzog AR (2005). Documentation of cognitive functioning measures in the Health and Retirement Study.

[39] Wechsler D (1981). Wechsler Adult Intelligence Scale-Revised.

[40] Crottaz-Herbette S, Anagnoson RT, Menon V (2004). Modality effects in verbal working memory: differential prefrontal and parietal responses to auditory and visual stimuli. Neuroimage.

[41] Rabbitt P, Diggle P, Holland F, McInnes L (2004). Practice and drop-out effects during a 17-year longitudinal study of cognitive aging. J Gerontol B Psychol Sci Soc Sci.

[42] Herzog AR, Rodgers WL, Schwarz N, Park DC, Knauper B, Sudman S (1999). Cognitive performance measures in survey research on older adults. Cognition, Aging, and Self-Reports.

[43] Stern Y (2009). Cognitive reserve. Neuropsychologia.

[44] Deary IJ, Corley J, Gow AJ, Harris SE, Houlihan LM, Marioni RE, Penke L, Rafnsson SB, Starr JM (2009). Age-associated cognitive decline. Br Med Bull.

[45] Perlmutter M, Nyquist L (1990). Relationships between self-reported physical and mental health and intelligence performance across adulthood. J Gerontol.

[46] Fritsch T, McClendon MJ, Smyth KA, Lerner AJ, Friedland RP, Larsen JD (2007). Cognitive functioning in healthy aging: the role of reserve and lifestyle factors early in life. Gerontologist.

[47] Wesnes K (2002). Assessing cognitive function in clinical trials: latest developments and future directions. Drug Discov Today.

[48] Makdissi M, Collie A, Maruff P, Darby DG, Bush A, McCrory P, Bennell K (2001). Computerised cognitive assessment of concussed Australian Rules footballers. Br J Sports Med.

[49] Kalbe E, Salmon E, Perani D, Holthoff V, Sorbi S, Elsner A, Weisenbach S, Brand M, Lenz O, Kessler J, Luedecke S, Ortelli P, Herholz K (2005). Anosognosia in very mild Alzheimer's disease but not in mild cognitive impairment. Dement Geriatr Cogn Disord.

[50] Saykin AJ, Wishart HA, Rabin LA, Santulli RB, Flashman LA, West JD, McHugh TL, Mamourian AC (2006). Older adults with cognitive complaints show brain atrophy similar to that of amnestic MCI. Neurology.

[51] Coons SJ, Gwaltney CJ, Hays RD, Lundy JJ, Sloan JA, Revicki DA, Lenderking WR, Cella D, Basch E (2009). Recommendations on evidence needed to support measurement equivalence between electronic and paper-based patient-reported outcome (PRO) measures: ISPOR ePRO Good Research Practices Task Force report. Value Health.

[52] Small BJ, Dixon RA, McArdle JJ (2011). Tracking cognition-health changes from 55 to 95 years of age. J Gerontol B Psychol Sci Soc Sci.

[53] Dixon RA, Wahlin A, Maitland SB, Hultsch DF, Hertzog C, Bäckman L (2004). Episodic memory change in late adulthood: generalizability across samples and performance indices. Mem Cognit.

[54] Vuorisalmi M, Lintonen T, Jylhä M (2006). Comparative vs global self-rated health: associations with age and functional ability. Aging Clin Exp Res.

[55] Jylhä M, Guralnik JM, Balfour J, Fried LP (2001). Walking difficulty, walking speed, and age as predictors of self-rated health: the women's health and aging study. J Gerontol A Biol Sci Med Sci.

[56] Jylhä M (2009). What is self-rated health and why does it predict mortality? Towards a unified conceptual model. Soc Sci Med.

[57] Heerwegh D (2003). Explaining response latencies and changing answers using client-side paradata from a web survey. Soc Sci Comput Rev.

